# Preparation of Cellulose Fiber Loaded with CuO Nanoparticles for Enhanced Shelf Life and Quality of Tomato Fruit

**DOI:** 10.3390/ma17122823

**Published:** 2024-06-10

**Authors:** Senthilkumar Palanisamy, Nandhana Varnan, Shanmugam Venkatachalam, Kumarakuru Kuppuswamy, Gayathri Devi Selvaraju, Devanesan Sanjeevi Ranjith Santhosh Kumar, Rajendran Kamalabai Selvakesavan, Gokul Bangaru, Devaraj Bharathi

**Affiliations:** 1 School of Biotechnology, Dr. G R Damodaran College of Science, Coimbatore 641014, Tamil Nadu, India; 2 Department of Biotechnology, Nehru Arts and Science College, Coimbatore 641105, Tamil Nadu, India; 3 Department of Food Processing Technology, PSG College of Arts and Science, Coimbatore 641014, Tamil Nadu, India; 4 Department of Biotechnology, KIT-Kalaignarkarunanidhi Institute of Technology, Coimbatore 641402, Tamil Nadu, India; 5 Department of Biotechnology PSGR Krishnammal College for Women, Coimbatore 641004, Tamil Nadu, India; 6 Department of Physics, Kongunadu Arts and Science College, Coimbatore 641029, Tamil Nadu, India; 7 School of Chemical Engineering, Yeungnam University, 280 Daehak-Ro, Gyeongsan 38541, Republic of Korea

**Keywords:** antifungal, cellulose fiber, CuO, cytotoxicity, tomato shelf life

## Abstract

The present study reports on the preparation of a cellulose fiber (CF) composite from *D. lutescens*, combined with copper oxide nanoparticles (DL@CF/CuO), to prolong the shelf life of tomatoes after harvest. The isolated cellulose fiber material was comprehensively characterized using XRD, FTIR, and FE-SEM analyses. The DLCF and DL@CF/CuO nanoparticles exhibited crystalline cellulose, as indicated by the XRD investigation. Both DLCF and DL@CF/CuO showed O-H and C-H FTIR spectra with identifiable vibrational peaks. The FE-SEM images depicted the dispersion of DL@CF/CuO-based fibers in a cellulose fiber matrix containing CuO nanoparticles. A 0.3% (wt/wt), a solution of DL@CF/CuO was coated onto the surface of early ripening tomato fruits. After a 25-day storage period at 25–29 °C and 85% RH, the results showed a significant extension in the shelf life of the tomato fruits, in line with changes in physiological properties and fruit quality. The extension of shelf life in tomato fruit epidermis treated with DL@CF/CuO was confirmed through FE-SEM analysis. L929 fibroblast cells were treated with the developed DL@CF/CuO nanocomposite, and no signs of toxicity were detected up to 75 µg/mL. Additionally, the DL@CF/CuO nanocomposite exhibited significant antifungal activity against *Aspergillus flavus*. In conclusion, this study provides novel insights for sustainable food security and waste control in the agricultural and food industries.

## 1. Introduction

Tomato (*Lycopersicon esculentum* L.) belongs to the Solanaceae family and is the primary vegetable crop grown year-round, achieving universal popularity over the last century [1]. According to a market study report, it was estimated that 79.52 million tons of tomatoes were produced globally in 2022. It is expected that between 2023 and 2028, there will be a compound annual growth rate (CAGR) of 3.8%, leading to the production of 99.46 million tons [2]. 

Tomatoes boast a beautiful appearance, a tasty flavor, and a wealth of nutrients, including healthy vitamins, carotenoid pigments, and phenolic compounds known for promoting health [3,4]. However, ripened tomatoes are susceptible to various challenges such as harvesting damage, microbial decay, and deterioration in quality, leading to economic losses [5]. To minimize the postharvest losses of tomatoes, various physical methods have been implemented, including postharvest precooling, refrigeration storage, postharvest heat treatment, modified environment packing, and the use of chemical preservatives, edible coatings, composite coatings, edible film coatings, polymer coatings and so forth [6,7,8]. In many nations and regions, food waste is exacerbated by insufficient facilities for perishable fruits and vegetables and inadequate control over transportation, according to food and agriculture organization (FAO) data from 2012 [7]. Moreover, consumers may also express caution about chemically preserved fruits and vegetables due to potential health risks [8]. To address these postharvest loss problems in tomatoes, scientists have accelerated research on next-generation alternative methods. Recent scientific investigations have reported that nano-material-based inventions and processes enhance the quality of perishable fruits [9]. 

Cellulose is a biological macromolecule extensively found in plant cell walls, particularly in plants that produce fiber. Cellulose has a fibrous structure and is composed of both crystalline and amorphous regions with a high aspect ratio and a dense network of hydrogen bonds [10]. Cellulose fibers have shown significant potential in fruit preservation applications including providing a defensive barrier against moisture, oxygen exposure, and microbial attacks. These properties contribute to extending the shelf life of fruits by delaying ripening, maintaining quality, and reducing physical damage during handling and transportation [11,12].

Pure cellulose fibers have drawbacks for fruit preservation, including low mechanical strength, potential water vapor transmission, and inadequate adhesion to fruit surfaces. Additionally, their stability under varying pH and temperature conditions, and the cost of large-scale production pose challenges [13,14,15]. However, composite materials containing various particle types and CuO nanoparticles have demonstrated a noticeable improvement in the quality and shelf life of fruits [16,17,18].

Several significant results have been developed regarding the use of cellulose fibers combined with copper oxide nanoparticles to create composite materials for packaging and coating fresh fruits and vegetables. However, there is currently no research on the application of cellulose fibers combined with copper oxide composite materials specifically for tomato packaging. The present investigation is designed to examine the effect of coating tomatoes with cellulose fiber combined with CuO composite material on the physiological changes that occur during their shelf life when stored at 25–29 °C. This investigation introduces a novel approach for employing cellulose fiber with CuO composite material in preserving postharvest tomatoes.

## 2. Materials and Methods

### 2.1. Materials

The *Dypsis lutescens* (Areca Palm) plant was collected from Thirumalayampalayam, (10.871012 N, 76.928612 E) Coimbatore, Tamilnadu, India in January 2023. The collected samples were brought to the laboratory in a paper cover. Tomatoes were purchased from a neighboring marketplace in Coimbatore, Tamilnadu, India, and the tomato fruits were visually categorized based on their size, color, and fungal contamination status. Ten tomatoes each were placed in one of these three groups: control, just DLCF, and DL@CF/CuO. When the tomato fruits were brought inside the laboratory, they were washed with double-distilled water and then left to dry naturally in the air at room temperature. Copper oxide (CuO) nanoparticles (CAS number:1317-38-0) were purchased from Sigma Aldrich, St. Louis, MI, USA. The *Aspergillus flavus* (MTCC 2798) fungal strain was purchased from the Microbial Type Culture Collection (MTCC) in Chandigarh, India. Ordinary mouse fibroblasts L929 were sourced from the National Center for Cell Science (NCCS) in Pune, India. All chemicals employed in this research were acquired from HiMedia, India, and were of analytical grade quality.

### 2.2. Gas Chromatography-Mass Spectrometry (GC-MS) Analysis

GC-MS was performed to determine and identify phytochemicals present in the aqueous *D. lutescens* extract using Agilent GC-7890A/MS5975C (Santa Clara, CA, USA) equipped with a mass spectrophotometer. The conditions were as follows: a column length of 30 m, film thickness of 0.25 mm, and a retention period of 0.25 microns. The initial temperature was set at 60 °C for 2 min, followed by a gradual increase of 10 °C per minute up to 300 °C, and maintained at this temperature for 6 min. The flow rate was 1 mL/min, volume 1 µL, split ratio 10:1, and the overall duration was 32 min. The injection port was set to 260 °C, and helium was used as the carrier gas. MassHunter Workstation software (version 5.4.2) was utilized for the spectrum analysis. The structure of mass spectral patterns was determined using the NIST library.

### 2.3. Isolation of Cellulose Fibers from D. lutescens (DLCF)

Cellulose fibers were isolated from *D. lutescens* (DLCF). The stem of *D. lutescens* was segmented into small sections, air-dried for two weeks under shade, and then washed with distilled water and subsequently dried in a hot air oven set at 110 °C for 12 h. Next, the powder underwent Soxhlet extraction with toluene and ethanol (2:1) for 22 h. After extraction, the fibers were heated in a solution of 0.7% NaClO_2_ (sodium chlorite) at 100 °C for 2 h to remove lignin. The pH was regulated using a 10% acetic acid solution. The resulting liquid was filtered, and the fibers were extensively washed with sodium bisulfate. They were then centrifuged five times with distilled water, and the resulting pellets were dried in a hot-air oven at 110 °C. 

The material was treated with a 17% (*w*/*v*) NaOH solution for further processing and placed in a hot air oven at 20 °C. The alkaline material was then neutralized with 10% acetic acid, washed with distilled water, and filtered. Finally, the resulting product was subjected to drying at 105 °C in a hot air oven, resulting in the extraction of crude cellulose. Crude cellulose was mixed with a solution of 80% acetic acid and 70% HNO_3_ (10:1) and heated at 110 °C for 10 min in a hot air oven. Once the treatment was complete, the mixture was cooled using distilled water. It was then washed with 99% ethanol and filtered twice, followed by two additional washes with distilled water. 

### 2.4. Preparation of DL@CF/CuO Composite 

The fabrication process of the cellulose fiber composite (DL@CF/CuO) involved a sequence of steps designed to ensure the creation of a high-quality material. Following purification, DLCF was dispersed in distilled water at a concentration of 1% by mass, forming a homogeneous suspension. The dispersion process was meticulously controlled using a magnetic stirrer to guarantee thorough mixing. Simultaneously, 0.1% *w*/*v* CuO was gradually introduced into the dispersion of cellulose fibers with stirring. This precise addition of CuO is crucial to achieving the desired properties of the final composite material. The composite dispersion was then transferred to a sonication vessel and subjected to sonication for 20 min at 1000 W. The ultrasonic treatment was carried out in an ice bath to avoid overheating of the sample; this sonication step is vital in achieving a uniform distribution of CuO throughout the cellulose fiber matrix. After the sonication process, the suspension underwent centrifugation at 10,000 rpm for 20 min, separating the solid components from the liquid supernatant. The collected solid material, known as the pellet, was rinsed three times with deionized water to effectively remove any residual impurities or unwanted substances. Following this purification, the composite material was air-dried and stored in a refrigerator, preserving its quality for future utilization.

### 2.5. Characterization Studies

#### 2.5.1. Fourier Transform Infrared Spectroscopy (FTIR) Analysis

The functional groups of the samples were characterized using a Shimadzu FTIR 8400S spectrophotometer (Neuchatel, Switzerland) using the KBr pellet method, covering the wave number range from 4000 to 600 cm^−1^.

#### 2.5.2. Field Emission Scanning Electron Microscopy (FE-SEM) 

The surface characteristics of the isolated DLCF and DL@CF/CuO were analyzed through FE-SEM (HITACHI SU3500, Tokyo, Japan) equipped with energy dispersive spectroscopy (EDS).

### 2.6. Effect of DL@CF/CuO Coating on Tomato Shelf Life and Physicochemical Characteristics

Before starting the coating procedures, the fruits were immersed in a 0.05% sodium hypochlorite solution for 2 min. Subsequently, all the fruits were dipped for 1 min in DLCF alone and DL@CF/CuO 0.3% (wt %) solutions, respectively. For the control group, the fruits were dipped in distilled water. After coating, the tomatoes were kept at 25–29 °C and 85% RH for 25 days. The fruits were analyzed daily for any visible changes and every 5, 10, 15, 20, and 25 days to assess the shelf life and various physicochemical attributes, including weight loss, firmness, pH, titratable acidity, ascorbic acid, lycopene content, and moisture level. Epidermis samples from treated, and control tomatoes were analyzed using FE-SEM at 0 and 25 days of storage to assess the tomato surface. 

### 2.7. Antifungal Activity

The antifungal efficacy of DL@CF/CuO was evaluated through a mycelium growth inhibition test. The DLCF and DL@CF/CuO at a concentration of 10 μg/mL were added into the Sabouraud dextrose agar (SDA) medium. The *A. flavus* fungus culture was placed in the center of the Petri dishes, and the plates were then incubated for 7 days at 30 °C in an incubator. The fungal growth was measured by confirming their mean radius. The untreated fungal culture served as the negative control. The fungal mycelium growth inhibition was measured using the formula shown below:

Inhibition (%) = 100 × (growth control − growth treatment)/growth control.

### 2.8. Cell Viability Assay

The cells were added to a minimum essential medium (MEM) supplemented with 10% fetal bovine serum (FBS). The cells were kept under consistent conditions at 37 °C, within an atmosphere of 5% CO_2_, 95% air, and 100% relative humidity. To separate confluent cells, 0.25% trypsin was added. Subsequently, the separated cells were introduced into the 96-well plate, with a density of 25,000 cells per well, and left to incubate for 24 h. Following the initial incubation, the medium was replaced with a new medium containing DLCF and DL@CF/CuO at concentrations of 5, 25, 75, and 100 µg/mL. Subsequently, 100 µL of MTT solution was added to the treated and control wells, and both underwent 6 h incubation at 37 °C. To solubilize the medium with 3-(4,5-Dimethylthiazol-2-yl)-2,5-diphenyltetrazolium bromide (MTT), 100 µL of DMSO was added after the medium formed formazan crystals. The mixture was assessed by quantifying the absorbance at 570 nm using a microplate reader, and the percentage of cell inhibition was determined using the subsequent formula:

Cell inhibition (%) = 100 × ((100 − Abs (sample))/Abs (control))

where Abs (sample) and Abs (control) are the absorbances of the tested and control samples.

The structural properties of the cells were examined using an inverted phase contrast microscope (TE-300; Nikon, Tokyo, Japan).

### 2.9. Statistical Analysis

The control, DLCF-treated, and DL@CF/CuO-treated tomatoes were examined in triplicate, and standard deviations were computed. To find any observable differences, the data were subjected to one-way ANOVA analysis using SPSS 18 software (SPSS Inc., Cary, NC, USA). Subsequently, the significant distinctions among the mean values of the samples were ascertained by employing Duncan’s multiple range test (DMRT) with a significance level set at *p* < 0.05. 

## 3. Results and Discussion

### 3.1. Gas Chromatography-Mass Spectrometry (GC-MS) Analysis

**Figure 1:**
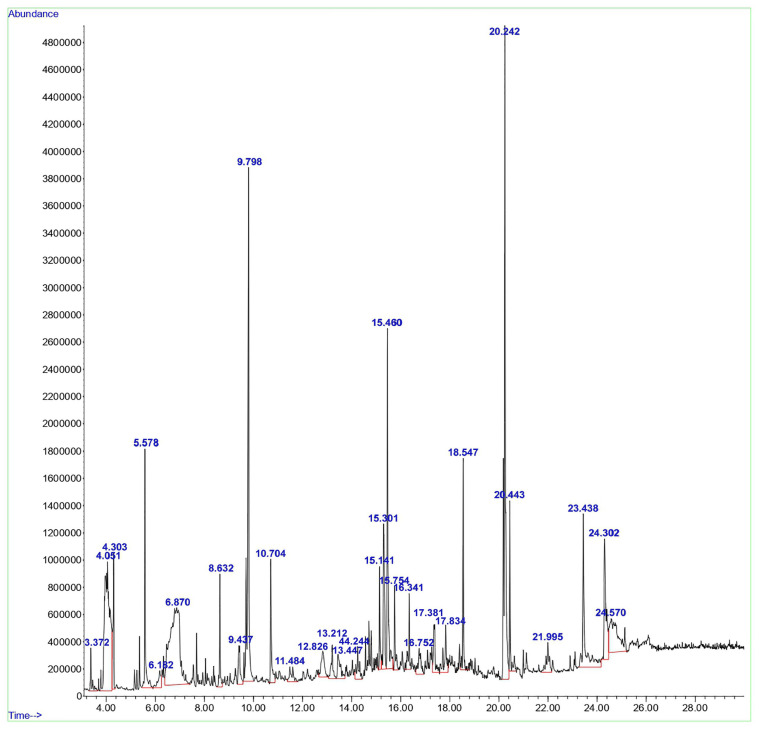
GC-MS analysis of the aqueous extract from the stem of *D. lutescens*.

GC-MS was employed to examine the compounds in the aqueous extract of *D. lutescens*. The chromatogram of the aqueous extract of *D. lutescens* is shown in Figure 1. This extract contains 30 plant compounds, which are displayed between retention times 2 and 30. The International Union of Pure and Applied Chemistry (IUPAC) name of the plant compounds, their molecular formulas, molecular weights, and applications are provided in Supplementary Table S1. The related compounds reported include 1,6-Anhydro-2,4-dideoxy-beta, 1-Decen-3-yne (9.437), (+-)-4-Amino-4,5-dihydro-2(3H)-furanone (4.303), and 2-Pentene, 3,4-dimethyl-, (E) (3.372). D-ribo-hexopyranose (12.826), 5-Benzylidene-4,5,6,7-tetrahydrobenzo[b]thiophen-4-one (24.302), (1R,3aS,5aS,8aR)-1,3a,4,5a-Tetramethyl-1,2,3,3a,5a,6,7,8-octahydrocyclopenta[c]pentalene (16.752), and 3H-Pyrazol-3-one, 1,2-dihydro-1,2,5-trimethyl- (13.212) showed no recorded activity.

Based on various studies [19,20,21,22], the anticancer properties of 2-Hexanol, 2-methyl- (4.051), Alpha.-Cadinol (15.301), Tyrosol, acetate (23.438), and Octadecanoic acid (20.443) have been investigated. 1,3-Dioxolane-4-methanol, 2,2-dimethyl- (5.578), Butane, 1,1-diethoxy- (6.182), and Nerolidol (14.244) exhibited antifungal activity [23]. Some compounds, such as glycerin (6.870), resorcinol (10.704), n-Hexadecanoic acid (18.547), cis-11-Hexadecenal (21.995), (1R,4S,5S)-1,8-Dimethyl-4-(prop-1-en-2-yl) spiro [4.5]dec-7-ene (15.141), Bicyclo [7.2.0]undec-4-ene, 4,11,11-trimethyl-8-methylene- (17.381), 1,3-Di(propen-1-yl)adamantane (17.834), and 9-Octadecenoic acid, (E)- (20.242), reported antimicrobial and antibiofilm activities [24,25,26,27,28,29,30,31]. The antioxidant properties were reported for 4H-Pyran-4-one, 2,3-dihydro-3,5-dihydroxy-6-methyl- (8.632), oxepine, and 2,7-dimethyl- (11.484) [32,33]. Two other compounds, 9,19-Cycloergost-24(28)-en-3-ol, 4,14-dimethyl-, (3β,4α,5α)- (24.570) and 7-Acetyl-2-hydroxy-2-methyl-5-isopropylbicyclo[4.3.0]nonane (16.341), were reported to have insecticidal activity [34,35]. 5-Hydroxymethylfurfural (9.798) showed antiallergic activity. Few other compounds, such as D-allose (13.447) and alpha-bisabolol (15.754), exhibited anti-inflammatory activity [35,36]. The (+)-epi-Bicyclosesquiphellandrene (15.301) was reported to have antidermatophytic activity [37,38,39,40].

### 3.2. Characterization of DLCF and DL@CF/CUO

**Figure 2:**
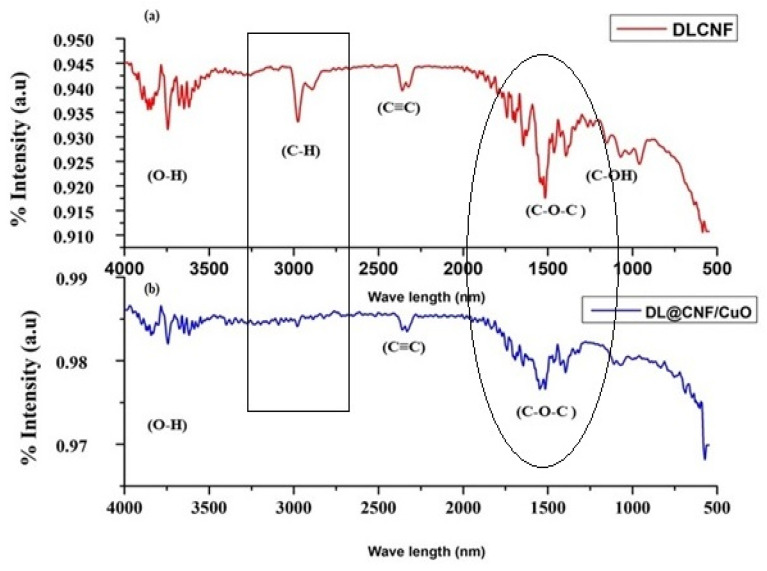
FTIR analysis of (**a**) DLCF and (**b**) DL@CF/CuO composite was carried out in the range between ~600 cm^−1^ and 4000 cm^−1^.

The FT-IR spectra of DLCF cellulose and DL@CF/CuO are presented in Figure 2a,b. The extracted cellulose from *D. lutescens* exhibited a prominent peak at 3827 cm^−1^, which was associated with the stretching vibration of O–H bonds, thereby confirming the hydrophilic nature of cellulose fibers (Figure 2a). C–H stretching and bending vibrations were assigned peaks at 2932 cm^−1^ and 2328 cm^−1^, respectively. The cellulosic structure’s C–O–C pyranose ring was confirmed by the peak at 1512 cm^−1^, and the peaks at approximately 1192 cm^−1^ and 1208 cm^−1^ might be attributed to glycosidic C–O–C and C–OH bonds, confirming the presence of carbohydrates [40]. Figure 2b exhibited the FT-IR results of DL@CF/CuO. In comparison to DLCF, the strength of O–H stretching at 3827 cm^−1^ was slightly decreased. This might be due to a decrease in the number of cellulose O–H groups, which react with copper ions to generate copper nanoparticles. In the presence of CuO deposited on cellulose, there was no peak at 2932 cm^−1^. This suggested that cellulose’s C–H groups and copper ions were interacting [41].

**Figure 3:**
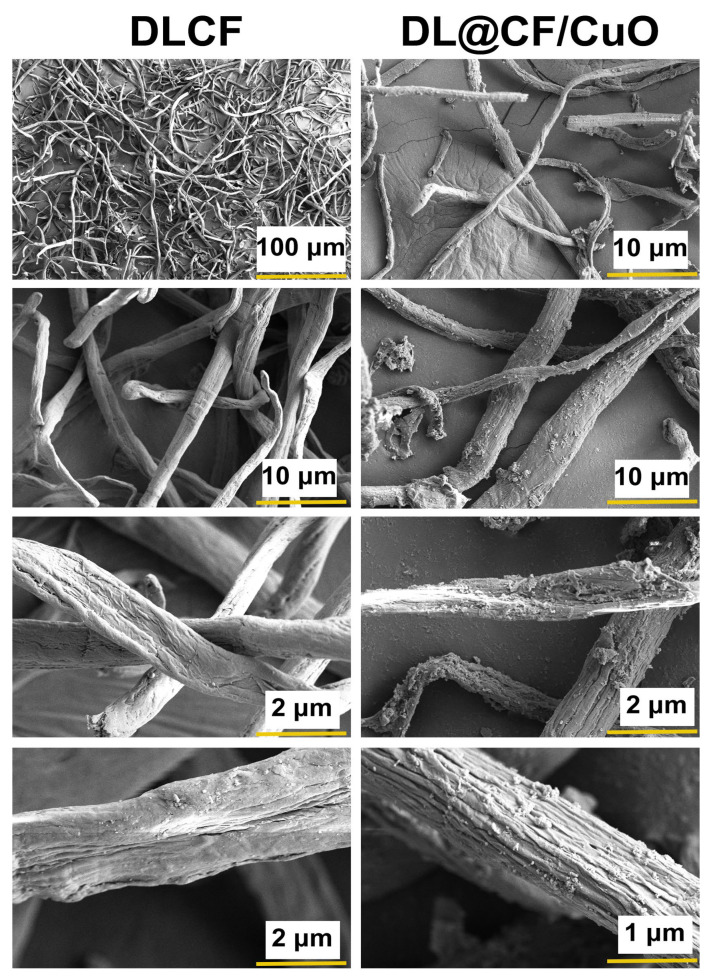
FE-SEM micrographs of the DLCF and DL@CF/CuO composite at different resolutions.

Figure 3 shows the FE-SEM images of DLCF and DL@CF/CuO. The FE-SEM analysis exhibited steady exclusion of amorphous chemical materials, and the images also showed that considerable cellulose was retained even after acid hydrolysis [42]. The distribution of DL@CF/CuO-based fibers in the cellulose fiber matrix with CuO nanoparticles has been observed in the FE-SEM images. CuO nanoparticles might have adhered to the surface of DLCF fibers via electrostatic adsorption when the fibers were submerged in a suspension of CuO nanoparticles [43,44,45].

### 3.3. Effect of DLCF and DL@CF/CuO Coating on Tomato Shelf Life and Physio-Chemical Characteristics

**Figure 4:**
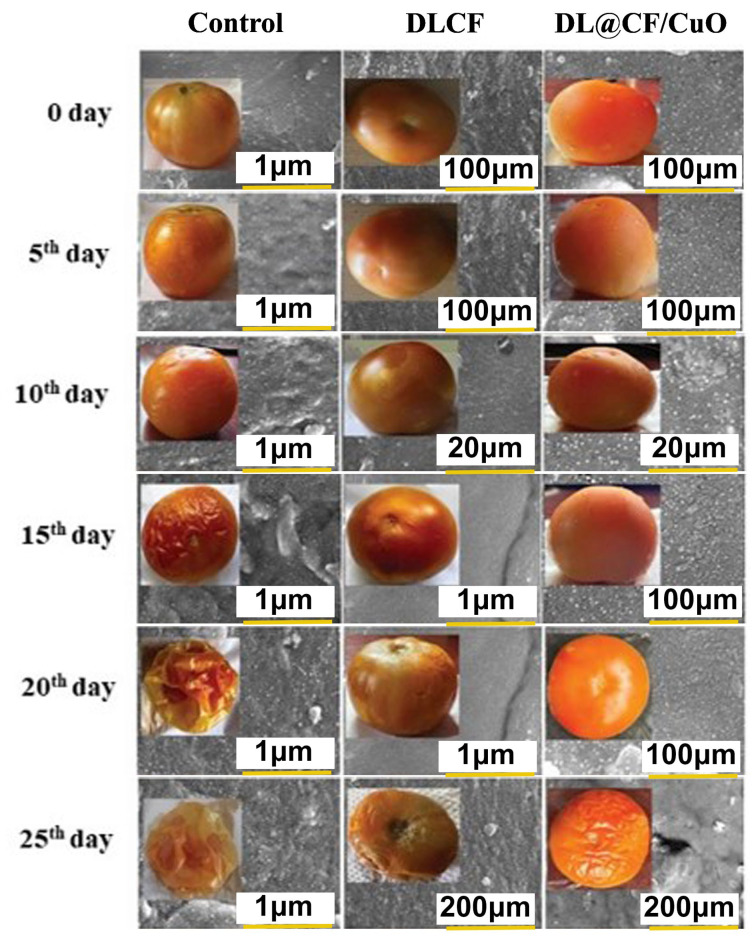
FE-SEM images of the epidermis of control and treated tomato fruit with DLCF and DL@CF/CuO are shown. The insets show images of tomato fruit treated with or without the DLCF and DL@CF/CuO composite at 25–29 °C and 85% RH over a 25-day storage period.

**Figure 5:**
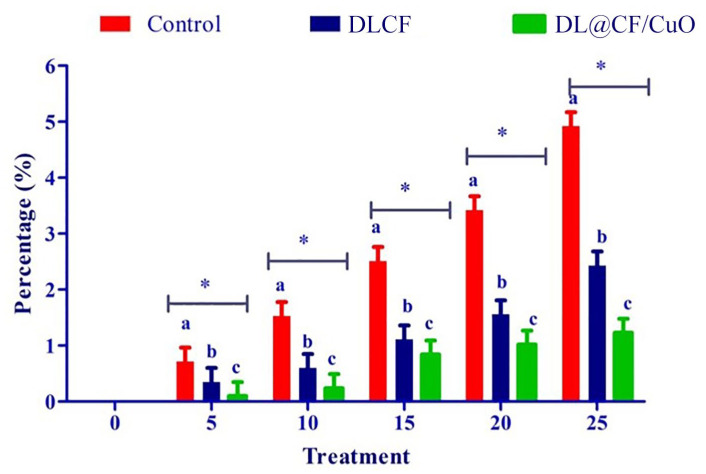
Effect of DLCF and DL@CF/CuO on weight loss of tomatoes stored at 25–29 °C. Each value is the mean of triplicates, and each replication consisted of five tomatoes. The bars represent the mean values with standard error. Groups labeled with different letters (a, b, c) are significantly different from each other (*p* < 0.05). The asterisk (*) indicates a statistically significant difference between the groups connected by the horizontal line.

To evaluate the weight loss throughout the storage of the tomatoes, control, DLCF, and DL@CF/CuO-treated tomatoes were weighed on days 0, 5, 10, 15, 20, and 25 after treatments. With the prolongation of the storage period for all treatments, the percentage of weight loss and moisture increased significantly (Figure 4 and Table 1). The untreated control group displayed a percentage of weight loss of 4.92% after 25 days of storage. The tomatoes treated with DLCF displayed a percentage of weight loss of 2.13%, while the tomatoes treated with DL@CF/CuO displayed a percentage of weight loss of 1.21% (Figure 5). The outcomes verified that both DLCF and DL@CF/CuO treatments notably decreased the weight loss percentage of tomatoes in comparison to the control group. The cellulose fibers possibly formed a shielding barrier around the tomatoes, reducing moisture loss throughout storage, while the combination of CuO nanoparticles in DL@CF/CuO-treated tomatoes contributed to added preservation properties through its antimicrobial properties. These findings highlight the potential of cellulose-incorporated nanoparticle-based materials for improving the shelf life and overall quality of tomatoes.

**Figure 6:**
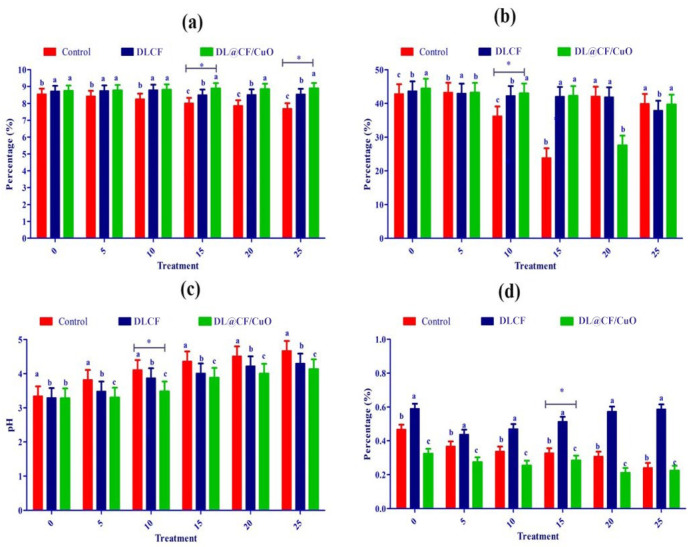
Effect of DLCF and DL@CF/CuO composition on (**a**) ascorbic acid, (**b**) firmness, (**c**) pH, and (**d**) titratable acidity of tomatoes stored at 25–29 °C. Each value represents the mean of triplicates, and each replication consisted of five tomatoes. Groups labeled with different letters (a, b, c) are significantly different from each other (*p* < 0.05). The asterisk (*) indicates a statistically significant difference between the groups connected by the horizontal line.

Fruit firmness was assessed utilizing a digital penetrometer, recording the force needed to penetrate the tomato’s surface at various evenly spaced spots (Figure 6a–d). The results showed that both DLCF and DL@CF/CuO treatments had a significant impact on tomato fruit firmness compared to the control group. Notably, the DL@CF/CuO treatment exhibited a greater enhancement in firmness than the DLCF treatment. The firmness of untreated tomatoes was markedly reduced on the 10th day of storage (23.82 ± 0.12 N) compared to DLCF-treated (42.02 ± 0.34 N) and DL@CF/CuO-treated (42.25 ± 0.11 N) tomatoes at 25–29 °C. When compared to the control, the higher firmness values of the DLCF- and DL@CF/CuO-treated tomatoes were significant (*p* < 0.05). These findings support the promise of cellulose-based treatments in maintaining postharvest quality and prolonging the shelf life of tomato fruits.

Over time, the pH and titratable acidity of tomato fruits were treated with or without DLCF and DL@CF/CuO (Figure 6c). Results indicate a pH increase in all the tomato samples; however, the control samples exhibited a higher increase than both DLCF and DL@CF/CuO samples. The DLCF- and DL@CF/CuO-treated tomatoes had higher and statistically significant pH values compared to control samples on the 10th day of storage at 25–29 °C. During the storage period, the pH value increased due to acid disintegration that occurs as respiration takes place. According to the study’s findings, the pH of tomatoes increased after storage, falling within the range shown in earlier studies [45].

Figure 6d demonstrated a notable interaction between treatments and storage duration concerning the titratable acidity of tomato fruits. The utilization of DLCF had no substantial impact on the titratable acidity of the tomatoes (0.51 ± 0.01). Despite this, on the 15th day of the storage period, the slightly reduced titratable acidity value in the control (0.62 ± 0.01) and DL@CF/CuO-treated tomatoes (0.58 ± 0.02) indicated that the cellulose fiber has a delayed ripening effect, blocking the tomato fruits’ ability to breathe at their normal rate [46]. The observed rise in pH corresponds directly with the decrease in titratable acidity, aligning with earlier results that this trend signifies a decline in acid concentrations as fruits ripen [47].

Ascorbic acid, an essential antioxidant compound, is found in large quantities in green tomatoes, but its concentration steadily decreases during the ripening process [48]. In the present investigation, ascorbic acid content gradually reduced over 25 days of storage in control tomatoes (7.68 ± 0.10 mg/100 g) compared to DLCF-treated (8.544 ± 0.13 mg/100 g) and DL@CF/CuO-treated (8.89 ± 0.10 mg/100 g) tomatoes (Figure 6a). This study confirmed that, when tomatoes were kept at temperatures between 25 and 29 °C, the presence of DL@CF/CuO resulted in a considerably lower reduction in ascorbic acid content compared to untreated tomatoes. Nevertheless, when contrasted with a previous investigation relating to cherry tomatoes stored in Cu-chitosan NPs, the amount of ascorbic acid was maintained [49].

Lycopene pigment is responsible for the typical red color found in tomatoes [50,51]. The significant effect of control, DLCF, and DL@CF/CuO treatments on tomato lycopene content over different storage days is shown in Table 2. On days 0 and 5, minimal variation was observed in the levels of tomato lycopene across the different treatments. However, by day 10, the lycopene content of fruits treated with DLCF (3.89 ± 0.01%) and DL@CF/CuO (3.27 ± 0.01%) was higher than that of the control tomatoes (3.90 ± 0.01%).

Tomato peel samples treated with DLCF and DL@CF/CuO showed noticeable structural changes at the nanoscale level, as shown in the FE-SEM analysis (Figure 6). In the case of DLCF treatment, FE-SEM images showed a consistent distribution of cellulose networks adhering to the peel surface, forming a thin and dense coating. This coating seemed to establish a protective barrier, potentially diminishing water loss and maintaining the integrity of the peel. On the other hand, DL@CF/CuO-treated tomato peels showed a more complex surface morphology. FE-SEM analysis revealed the presence of CuO nanoparticles embedded within the cellulose fiber network. This network structure enhances the antimicrobial and antioxidant activity, which could significantly extend the shelf life of tomato fruits [52,53]. A similar type of microstructure of cellulose fiber films from carrot pomace was reported by Amoroso et al. [54]. The FE-SEM results revealed that both DLCF and DL@CF/CuO treatments encourage changes in the tomato peel microstructure, with DL@CF/CuO exhibiting an added nanoparticle-mediated effect.

### 3.4. Antifungal Activity

**Figure 7:**
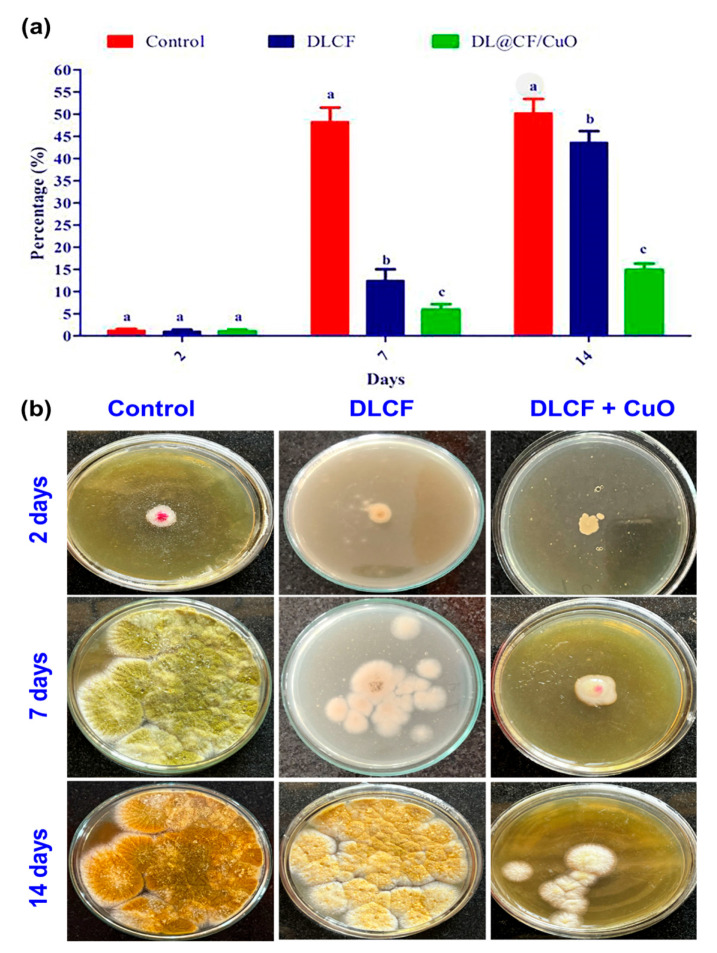
Antifungal activity of control, DLCF and DL@CF/CuO composite against the radial mycelial growth of *A. flavus*. (**a**) The percentage of mycelial growth was plotted in a histogram, and (**b**) images of control and treated fungal cultures were taken on the 2nd, 7th, and 14th day. Groups labeled with different letters (a, b, c) are significantly different from each other (*p* < 0.05).

The current study evaluates the antifungal activity depicted in Figure 7. The amount of fungal development in the control tomato samples was very high. In contrast, the tomato samples that were subjected to DLCF showed a significant decrease in the growth of fungal mycelia, indicating that DLCF may have the ability to prevent fungal growth. However, the tomato samples treated with DL@CF/CuO showed the most encouraging findings, confirming the lowest amount of fungal mycelia growth across all evaluated conditions. This tendency was evident on day 2, 7, and 14 of the posttreatment period, signifying the consistent and long-term antifungal efficiency of DL@CF/CuO treatment. This study emphasizes the prospective antifungal characteristics of DL@CF/CuO. These findings are in line with earlier research conducted by Bhardwaj et al. [55] and Bhavyasree et al. [56], which demonstrated the antifungal activity of copper nanoparticle-coated nanomaterials in agricultural applications, supporting the view that nanomaterials can be harnessed as efficient antifungal agents in fruit preservation strategies.

### 3.5. Cell Viability Assay

**Figure 8:**
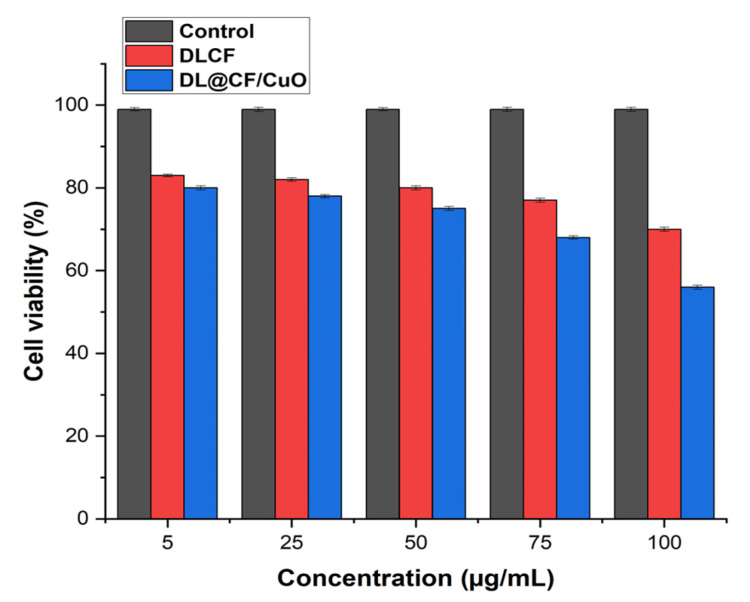
Bar diagram representing the cell viability assay of L929 cells treated with 5, 25, 75, and 100 μg/mL concentrations of DLCF and DL@CF/CuO.

L929 murine fibroblasts were used in a cell viability assay to investigate the cytotoxic effects of DLCF- and DL@CF/CuO-treated tomatoes at various concentrations, as shown in Figure 8 and Figure S1. The DLCF and DL@CF/CuO at concentrations ranging from 5 to 75 µg/mL demonstrated no significant differences in cell viability from the control group. Nevertheless, cell viability was shown to slightly decline at the highest concentration of 100 µg/mL. These findings suggest that DL@CF/CuO, up to a certain concentration, has minimal cytotoxic effects on L929 mouse fibroblasts. Similarly, when epidermal cells were used to evaluate silver-nanoparticles/bacterial cellulose composites, a low level of cytotoxicity was observed [46]. The in vitro cell viability investigation revealed that the cytotoxicity of DL@CF/CuO was comparatively low. According to this study, DL@CF/CuO material might be considered a feasible and safe approach for treating tomatoes to extend their shelf life.

## 4. Conclusions

In conclusion, this study presents new advances relating to the utilization of a composite material mixture of cellulose fibers synergistically embedded with CuO nanoparticles as a DL@CF/CuO composite to enhance the postharvest shelf life and quality of the tomato fruit. A key feature of this study encompassed the process of cellulose isolation and successive characterization. The isolation procedure carefully involved mechanical homogenization and chemical treatment, followed by the release of cellulose fibers from *D. lutescens*. Characterization techniques such as XRD, FTIR, and FE-SEM were employed to analyze the unique nanostructure, which extensively influences interactions with the tomato fruit matrix. Furthermore, the promising outcomes unveiled the potential of the cellulose fiber material to significantly delay the ripening process by preserving critical physicochemical parameters in the tomato fruit. This study showcases the innovative addition of cutting-edge nanotechnological agricultural practices. Hence, this investigation provides an effective fruit preservative cellulose material with high competence.

## Figures and Tables

**Table 1:** Effect of moisture content on tomato fruit treated with DLCF and DL@CF/CuO composite during storage.

Samples	Treatments		
	Control	DLCF	DL@CF/CuO
0	92.11 ± 0.42 ^b^	94.34 ± 0.40 ^a^	94.44 ± 0.55 ^a^
5	91.44 ± 0.38 ^b^	94.23 ± 0.47 ^a^	93.08 ± 0.51 ^a^
10	91.53 ± 0.52 ^b^	94.60 ± 0.48 ^a^	93.36 ± 0.52 ^a^
15	82.34 ± 0.33 ^b^	93.11 ± 0.42 ^a^	94.84 ± 0.53 ^a^
20	80.25 ± 0.30 ^c^	90.30 ± 0.41 ^b^	94.02 ± 0.41 ^a^
25	78.90 ± 0.40 ^c^	90.10 ± 0.45 ^b^	93.63 ± 0.42 ^a^

Data are expressed as mean ± standard deviation of triplicates. Means within the same row that have no common superscripts are significantly different. Groups labeled with different letters (a, b, c) are significantly different from each other (*p* < 0.05).

**Table 2:** Effect of lycopene content on tomato fruit treated with control, DLCF, and DL@CF/CuO during storage.

Samples	Treatments		
	Control	DLCF	DL@CF/CuO
0	2.23 ± 0.00 ^a^	2.24 ± 0.00 ^b^	2.10 ± 0.00 ^c^
5	2.97 ± 0.01 ^c^	3.35 ± 0.01 ^a^	2.34 ± 0.01 ^b^
10	3.90 ± 0.01 ^b^	3.89 ± 0.01 ^a^	3.27 ± 0.01 ^c^
15	4.46 ± 0.01 ^b^	5.45 ± 0.02 ^a^	3.95 ± 0.01 ^c^
20	5.12 ± 0.01 ^b^	6.67 ± 0.02 ^a^	4.28 ± 0.01 ^c^
25	6.44 ± 0.01 ^b^	6.81 ± 0.05 ^a^	7.73 ± 0.02 ^c^

Data are expressed as mean ± standard deviation of triplicates. Means within the same row that have no common superscripts are significantly different. Groups labeled with different letters (a, b, c) are significantly different from each other (*p* < 0.05).

## Data Availability

The raw data supporting the conclusions of this article will be made available by the authors on request.

## Supplementary Materials

The following supporting information can be downloaded at: https://www.mdpi.com/article/10.3390/ma17122823/s1, Table S1: GC-MS spectral analysis of aqueous extract of *D. lutescens*. Figure S1: Microscopic analysis of the cell morphology of L929 cells treated with DLCF, DL@CF/CuO and control, respectively.

## References

[1] Quinet M., Angosto T., Yuste-Lisbona F.J., Blanchard-Gros R., Bigot S., Martinez J.-P., Lutts S. (2019). Tomato Fruit Development and Metabolism. Front. Plant Sci..

[2] Tomato Processing Market Size, Share, Analysis, Forecast 2023–2028. https://www.expertmarketresearch.com/reports/tomato-processingmarket.

[3] Raiola A., Rigano M.M., Calafiore R., Frusciante L., Barone A. (2014). Enhancing the health-promoting effects of tomato fruit for biofortified food. Mediat. Inflamm..

[4] Martí R., Roselló S., Cebolla-Cornejo J. (2016). Tomato as a source of carotenoids and polyphenols targeted to cancer prevention. Cancers.

[5] Arah I.K., Ahorbo G.K., Anku E.K., Kumah E.K., Amaglo H. (2016). Postharvest Handling Practices and Treatment Methods for Tomato Handlers in Developing Countries: A Mini Review. Adv. Agric..

[6] Ali A., Yeoh W.K., Forney C., Siddiqui M.W. (2018). Advances in postharvest technologies to extend the storage life of minimally processed fruits and vegetables. Crit. Rev. Food Sci. Nutr..

[7] Kumar A., Saini C.S. (2021). Edible composite bi-layer coating based on whey protein isolate, xanthan gum and clove oil for prolonging shelf life of tomatoes. Meas. Food.

[8] Mondal K., Bhattacharjee S.K., Ghosh C., Vaibhav V.V., Katiyar V. (2022). Development of antioxidant-rich edible active films and coatings incorporated with de-oiled ethanolic green algae extract: A candidate for prolonging the shelf life of fresh produce. RSC Adv..

[9] Ala M.A.N., Shahbazi Y. (2019). The effects of novel bioactive carboxymethyl cellulose coatings on food-borne pathogenic bacteria and shelf life extension of fresh and sauced chicken breast fillets. LWT.

[10] FAO (2012). Save Food: Global Initiative on Food Loss and Waste Reduction.

[11] Palumbo M., Attolico G., Capozzi V., Cozzolino R., Corvino A., de Chiara M.L.V., Pace B., Pelosi S., Ricci I., Romaniello R. (2022). Emerging Postharvest Technologies to Enhance the Shelf-Life of Fruit and Vegetables: An Overview. Foods.

[12] Sridhar A., Ponnuchamy M., Kumar P.S., Kapoor A. (2021). Food preservation techniques and nanotechnology for increased shelf life of fruits, vegetables, beverages and spices: A review. Environ. Chem. Lett..

[13] Mokhena T.C., John M.J. (2020). Cellulose Nanomaterials: New Generation Materials for Solving Global Issues. Cellulose.

[14] Pirozzi A., Ferrari G., Donsì F. (2021). The use of nanocellulose in edible coatings for the preservation of perishable fruits and vegetables. Coatings.

[15] Huang L., Sun D.-W., Pu H., Zhang C., Zhang D. (2023). Nanocellulose-based polymeric nanozyme as bioinspired spray coating for fruit preservation. Food Hydrocoll..

[16] Saedi S., Garcia C.V., Kim J.T., Shin G.H. (2021). Physical and chemical modifications of cellulose fibers for food packaging applications. Cellulose.

[17] Liu Y., Ahmed S., Sameen D.E., Wang Y., Lu R., Dai J., Li S., Qin W. (2021). A review of cellulose and its derivatives in biopolymer-based for food packaging application. Trends Food Sci. Technol..

[18] Ai B., Zheng L., Li W., Zheng X., Yang Y., Xiao D., Shi J., Sheng Z. (2021). Biodegradable Cellulose Film Prepared from Banana Pseudo-Stem Fibers for Mango Preservation. Front. Plant Sci..

[19] Amara C., El Mahdi A., Medimagh R., Khwaldia K. (2021). Nanocellulose-based composites for packaging apppplications. Curr. Opin. Green Sustain. Chem..

[20] Lakshmi P., Babu S., Arulselvan S., Selvam P. (2022). Effect of CuO nanoparticles on the shelf life of guava (*Psidium guajava*) fruits and juice. J. Food Sci. Technol..

[21] López-Vargas G., García-Gómez M., Vázquez-Rodríguez A., Martínez-Hernández P. (2018). Copper oxide nanoparticles for the improvement of the postharvest quality of tomatoes. Food Sci. Technol..

[22] Jalill R.D.A. (2018). Chemical analysis and anticancer effects of *Juniperus polycarpos* and oak gall plants extracts. Res. J. Pharm. Technol..

[23] Su Y.-C., Hsu K.-P., Wang E.I.-C., Ho C.-L. (2013). Composition and in vitro Anticancer activities of the leaf essential oil of *Neolitsea variabillima* from Taiwan. Nat. Prod. Commun..

[24] Vlachogianni I.C., Fragopoulou E., Kostakis I.K., Antonopoulou S. (2015). In vitro assessment of antioxidant activity of tyrosol, resveratrol and their acetylated derivatives. Food Chem..

[25] Yu F., Lu S., Yu F., Shi J., McGuire P.M., Wang R. (2008). Cytotoxic activity of an octadecenoic acid extract from *Euphorbia kansui* (Euphorbiaceae) on human tumour cell strains. J. Pharm. Pharmacol..

[26] Lee S.-J., Han J.-I., Lee G.-S., Park M.-J., Choi I.-G., Na K.-J., Jeung E.-B. (2007). Antifungal effect of eugenol and nerolidol against *Microsporum gypseum* in a guinea pig model. Biol. Pharm. Bull..

[27] Nalawade T.M., Bhat K., Sogi S.H.P. (2015). Bactericidal activity of propylene glycol, glycerine, polyethylene glycol 400, and polyethylene glycol 1000 against selected microorganisms. J. Int. Soc. Prev. Community Dent..

[28] Molinelli E., Brisigotti V., Simonetti O., Sapigni C., D’Agostino G.M., Rizzetto G., Giacchetti A., Offidani A. (2022). Efficacy and safety of topical resorcinol 15% versus topical clindamycin 1% in the management of mild-to-moderate hidradenitis suppurativa: A retrospective study. Dermatol. Ther..

[29] Omotoyinbo B.I., Afe A.E., Kolapo O.S., Alagbe O.V. (2018). Bioactive constituents of essential oil from Khaya senegalensis (Desr.) bark extracts. Am. J. Chem. Biochem. Eng..

[30] Xie J., Li X., Li W., Ding H., Yin J., Bie S., Li F., Tian C., Han L., Yang W. (2023). Characterization of the key volatile organic components of different parts of fresh and dried perilla frutescens based on headspace-gas chromatography-ion mobility spectrometry and headspace solid phase microextraction-gas chromatography-mass spectrometry. Arab. J. Chem..

[31] Singh V.K., Kavita K., Prabhakaran R., Jha B. (2013). Cis-9-octadecenoic acid from the rhizospheric bacterium Stenotrophomonas maltophilia BJ01 shows quorum quenching and anti-biofilm activities. Biofouling.

[32] Iwu M., Ezeugwu C., Okunji C., Sanson D.R., Tempesta M.S. (1990). Antimicrobial Activity and Terpenoids of the Essential Oil of hyptis Suaveolens. Int. J. Crude Drug Res..

[33] Qader K.O., Al-Saadi S.A.M., Faraj I.M. (2018). Phytochemical Constituents of Leaves Essential oils of *Achillea fragrantissima* (Asteraceae) from Iraq. Aro Sci. J. KOYA Univ..

[34] Yu X., Zhao M., Liu F., Zeng S., Hu J. (2013). Identification of 2,3-dihydro-3,5-dihydroxy-6-methyl-4H-pyran-4-one as a strong antioxidant in glucose–histidine Maillard reaction products. Food Res. Int..

[35] Subashri B., Pillai Y. (2014). J. A comparative study of antioxidant activity of *Bacopa monnieri* (L.) Pennell using various solvent extracts and its GC-MS analysis. Int. J. Pharm. Pharm. Sci..

[36] Kashere M.A., Tijjani A., Sabo M.U., Aliyu M. (2023). Evaluation of phytochemical compounds in ficus polita leaves powders for insect pest control. J. Agripreneurship Sustain. Dev..

[37] Shanti V.C.N., Neerakkal I. (2021). GC-MS analysis and in silico activity prediction of phytocompounds in the roots of *Chrysopogon zizanioides* (L.) Roberty. Plant Sci. Today.

[38] Gao D., Kawai N., Nakamura T., Lu F., Fei Z., Tamiya T. (2013). Anti-inflammatory effect of D-allose in cerebral ischemia/reperfusion injury in rats. Neurol. Med. Chir..

[39] Kamatou G.P.P., Viljoen A.M. (2010). A Review of the application and pharmacological properties of *α*-bisabolol and *α*-bisabolol-rich oils. J. Am. Oil Chem. Soc..

[40] Kuiate J.-R., Bessière J.M., Zollo P.H.A., Kuate S.P. (2006). Chemical composition and antidermatophytic properties of volatile fractions of hexanic extract from leaves of Cupressus lusitanica Mill. from Cameroon. J. Ethnopharmacol..

[41] Pang Q., Wang L., Yang H., Jia L., Pan X., Qiu C. (2014). Cellulose-derived carbon bearing –Cl and –SO3H groups as a highly selective catalyst for the hydrolysis of cellulose to glucose. RSC Adv..

[42] French A.D. (2014). Idealized powder diffraction patterns for cellulose polymorphs. Cellulose.

[43] Morán J.I., Alvarez V.A., Cyras V.P., Vázquez A. (2008). Extraction of cellulose and preparation of nanocellulose from sisal fibers. Cellulose.

[44] Tomšič B., Marković D., Janković V., Simončič B., Nikodinovic-Runic J., Ilic-Tomic T., Radetić M. (2022). Biodegradation of cellulose fibers functionalized with CuO/Cu2O nanoparticles in combination with polycarboxylic acids. Cellulose.

[45] Tibolla H., Pelissari F., Martins J., Vicente A., Menegalli F. (2018). Cellulose nanofibers produced from banana peel by chemical and mechanical treatments: Characterization and cytotoxicity assessment. Food Hydrocoll..

[46] Wu J., Zheng Y., Song W., Luan J., Wen X., Wu Z., Chen X., Wang Q., Guo S. (2014). In situ synthesis of silver-nanoparticles/bacterial cellulose composites for slow-released antimicrobial wound dressing. Carbohydr. Polym..

[47] Dong F., Li S., Liu Z., Zhu K., Wang X., Jin C. (2015). Chunde, Improvement of quality and shelf life of strawberry with nanocellulose/chitosan composite coatings. Bangladesh J. Bot..

[48] Sinha S.R., Singha A., Faruquee M., Jiku A.S., Rahaman A., Alam A., Kader M.A. (2019). Post-harvest assessment of fruit quality and shelf life of two elite tomato varieties cultivated in Bangladesh. Bull. Natl. Res. Cent..

[49] Meena M., Pilania S., Pal A., Mandhania S., Bhushan B., Kumar S., Gohari G., Saharan V. (2020). Cu-chitosan nano-net improves keeping quality of tomato by modulating physio-biochemical responses. Sci. Rep..

[50] Anthon G.E., LeStrange M., Barrett D.M. (2011). Changes in pH, acids, sugars and other quality parameters during extended vine holding of ripe processing tomatoes. J. Sci. Food Agric..

[51] Steelheart C., Alegre M.L., Baldet P., Rothan C., Bres C., Just D., Okabe Y., Ezura H., Ganganelli I., Grozeff G.E.G. (2020). The effect of low ascorbic acid content on tomato fruit ripening. Planta.

[52] Kaewklin P., Siripatrawan U., Suwanagul A., Lee Y.S. (2018). Active packaging from chitosan-titanium dioxide nanocomposite film for prolonging storage life of tomato fruit. Int. J. Biol. Macromol..

[53] Frusciante L., Carli P., Ercolano M.R., Pernice R., Di Matteo A., Fogliano V., Pellegrini N. (2007). Antioxidant nutritional quality of tomato. Mol. Nutr. Food Res..

[54] Amoroso L., De France K.J., Milz C.I., Siqueira G., Zimmermann T., Nyström G. (2021). Sustainable cellulose nanofiber films from carrot pomace as sprayable coatings for food packaging applications. ACS Sustain. Chem. Eng..

[55] Bhardwaj S., Lata S., Garg R. (2023). Application of nanotechnology for preventing postharvest losses of agriproducts. J. Hortic. Sci. Biotechnol..

[56] Bhavyasree P., Xavier T. (2022). Green synthesised copper and copper oxide based nanomaterials using plant extracts and their application in antimicrobial activity: Review. Curr. Res. Green. Sustain. Chem..

