# Supplementary material for: Preparation of Cellulose Fiber Loaded with CuO Nanoparticles for Enhanced Shelf Life and Quality of Tomato Fruit
Source: Materials (Basel). 2024 Jun 10;17(12):2823. doi: 10.3390/ma17122823 (PMC11204958; doi:10.3390/ma17122823)
Supplement: Supplementary file 1 [file materials-17-02823-s001.zip › materials-2999830-supplementary.pdf]

SUPPLEMENTARY MATERIAL

Preparation of Cellulose Fiber Loaded with CuO Nanoparticles for Enhanced Shelf Life and Quality of Tomato Fruit

Senthilkumar Palanisamy [1,*], Nandhana Varnan [2], Shanmugam Venkatachalam [2], Kumarakuru Kuppuswamy [3],
Gayathri Devi Selvaraju [4], Devanesan Sanjeevi Ranjith Santhosh Kumar [5], Rajendran Kamalabai Selvakesavan [5], Gokul Bangaru [6] and Devaraj Bharathi [7,*]

[1] School of Biotechnology, Dr. G R Damodaran College of Science, Coimbatore 641014, Tamil Nadu, India
[2] Department of Biotechnology, Nehru Arts and Science College, Coimbatore 641105, Tamil Nadu, India; nandhanavarnan2001@gmail.com (N.V.); nasccentreresearch@nehrucolleges.com (S.V.)
[3] Department of Food Processing Technology, PSG College of Arts and Science, Coimbatore 641014, Tamil Nadu, India; kkumaraguru@psgcas.ac.in
[4] Department of Biotechnology, KIT- Kalaignarkarunanidhi Institute of Technology, Coimbatore 641402, Tamil Nadu, India; gayu.bt14@gmail.com
[5] Department of Biotechnology PSGR Krishnammal College for Women, Coimbatore 641004, Tamil Nadu, India; ranjith.deva87@gmail.com (D.S.R.S.K.); kesavanrks@gmail.com (R.K.S.)
[6] Department of Physics, Kongunadu Arts and Science College, Coimbatore 641029, Tamil Nadu, India; gokulbangaru@gmail.com
[7] School of Chemical Engineering, Yeungnam University, 280 Daehak-Ro, Gyeongsan 38541, Republic of Korea
* Correspondence: senthilkumar1185@gmail.com (S.P.); bharathi.deva@yahoo.in (D.B.)

Table S1:GC-MS spectral analysis of aqueous extract of *D. lutescens*

Peak	R. Time	IUPAC name	Molecular formula	Molecular weight	Nature/reported activity
1	3.372	2-Pentene, 3,4-dimethyl-, (E)	C_7H_{14}	98.19	No activity reported
2	4.051	2-Hexanol, 2-methyl-	$C_7H_{16}O$	116.20	Anticancer activity
3	4.303	(+-)-4-Amino-4,5-dihydro-2(3H)-furanone	$C_4H_7NO_2$	129.158	No activity reported
4	5.578	1,3-Dioxolane-4-methanol, 2,2-dimethyl-	$C_6H_{12}O_3$	132.16	Antifungal activity
5	6.182	Butane, 1,1-diethoxy-	C8H18O2	146.2273	Antifungal activity
6	6.870	Glycerin	$C_3H_8O_3$	*92.09*	Antimicrobial activity
7	8.632	4H-Pyran-4-one, 2,3-dihydro-3,5-dihydroxy-6-methyl-	$C_6H_8O_4$	144.1253	Antioxidant activity
8	9.437	1-Decen-3-yne	$C_{10}H_{16}$	136.23404	No activity reported
9	9.798	5-Hydroxymethylfurfural	$C_6H_6O_3$	126.11	Anti-allergic, anti-hypoxic
10	10.704	Resorcinol	$C_6H_6O_2$	110.1	Antimicrobial, Keratolytic activity
11	11.484	Oxepine, 2,7-dimethyl-	$C_8H_{10}O$	122.16	Antioxidant activity
12	12.826	1,6-Anhydro-2,4-dideoxy-. beta. -D-ribo-hexopyranose	C6H10O3	130.142	No activity reported.

13	13.212	3H-Pyrazol-3-one, 1,2-dihydro-1,2,5-trimethyl-	$C_{11}H_{13}ClN_2O_5$	288.68	No activity reported.
14	13.447	D-Allose	$C_6H_{12}O$	180.1559	Anti-inflammatory,anti-oxyradical effects
15	14.244	Nerolidol	*C15H26O*	222.37	Antifungal activity
16	15.141	(1R,4S,5S)-1,8-Dimethyl-4-(prop-1-en-2-yl) spiro [4.5] dec-7-ene	*C15H*	204.3511	Antibacterial activity
17	15.301	(+)-epi-Bicyclosesquiphellandrene	$C_{15}H_{24}$	204.3511	Antidermatophytic activity
18	15.460	Alpha. -Cadinol	$C_{15}H_{26}O$	222.37	Anticancer activity
19	15.754	alpha. -Bisabolol	$C_{15}H_{26}O$	284.7	Anti-inflammatory, anti-cancer, analgesic
20	16.341	7-Acetyl-2-hydroxy-2-methyl-5-isopropylbicyclo [4.3.0] nonane	$C_{15}H_{26}O_2$	238.366	anti-ulcer, analgesic and insecticidal activities
21	16.752	(1R,3aS,5aS,8aR)-1,3a,4,5a-Tetramethyl-1,2,3,3a,5a,6,7,8-octahydrocyclopenta[c]pentalene	$C_{15}H_{24}$	204.3511	No activity reported
22	17.381	Bicyclo[7.2.0]undec-4-ene, 4,11,11-trimethyl-8-methylene-	$C_{15}H_{24}$	204.3511	Antimicrobial activity
23	17.834	1,3-Di(propen-1-yl) adamantane	*C16H26*	216.36	Antimicrobial
24	18.547	n-Hexadecanoic acid	$C_{18}H_{36}O_2$	282.5	Antibacterial
25	20.242	9-Octadecenoic acid, (E)-			Anti-biofilm activity

26	20.443	Octadecanoic acid	$C_{18}H_{36}O_2$	284.4772	Cytotoxic activity
27	21.995	cis-11-Hexadecenal	$C_{16}H_{30}O$	238.4088	Antimicrobial
28	23.438	Tyrosol, acetate	$C_{10}H_{12}O_3$	180.2005	Anticancer activity
29	24.302	5-Benzylidene-4,5,6,7-tetrahydrobenzo[b]thiophen-4-one			No activity reported.
30	24.570	9,19-Cycloergost-24(28)-en-3-ol, 4,14-dimethyl-, (3. beta.,4. alpha.,5. alpha.)-	$C_{32}H_{52}O_2$	468.7541	Insecticidal activity

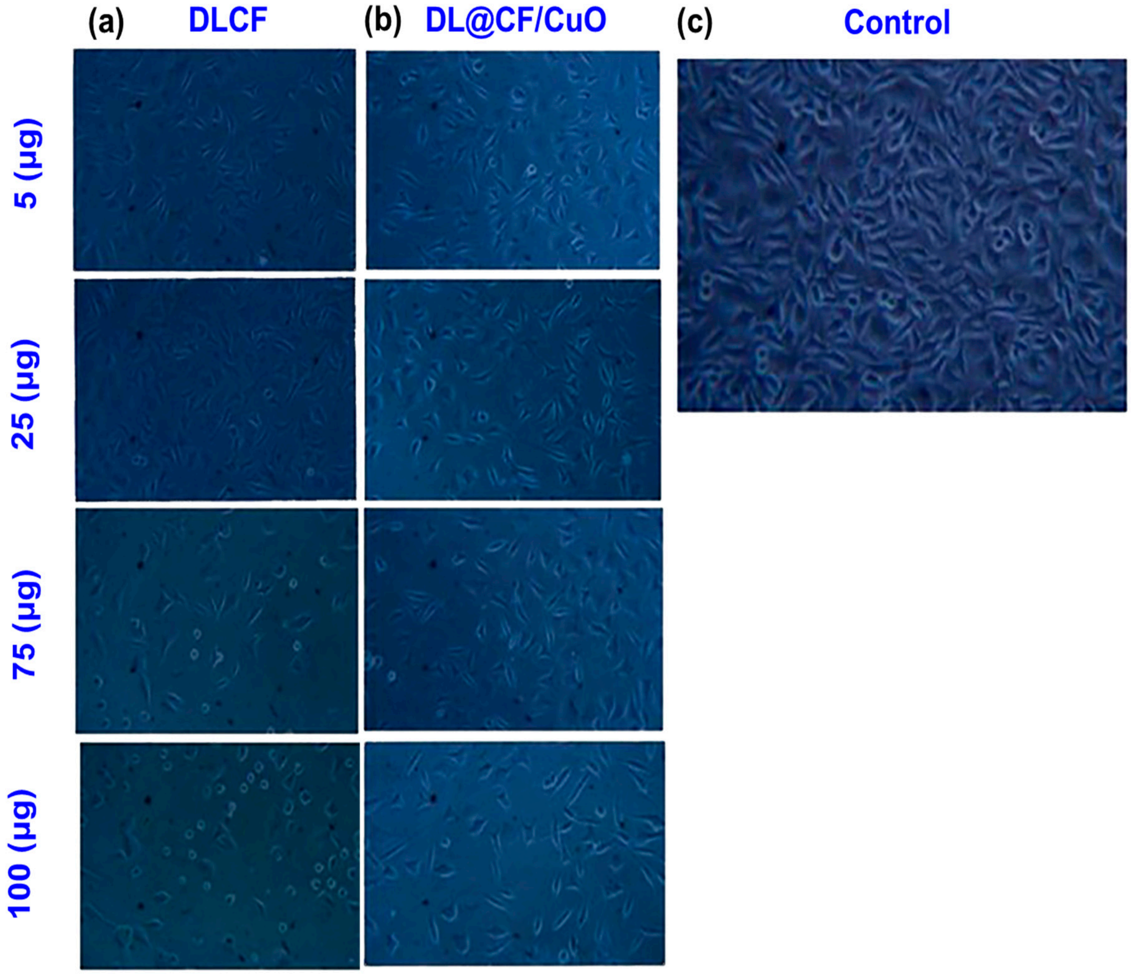

Figure S1. Microscopic analysis of the cell morphology of L929 cells treated with DLCF (a), DL@CF/CuO (b) and control (c), respectively.
